# Expanding benefits from cardiac resynchronization therapy to exercise‐induced left bundle branch block in advanced heart failure

**DOI:** 10.1002/ehf2.12580

**Published:** 2020-01-10

**Authors:** Fernando L. Scolari, Anderson D. Silveira, Willian R. Menegazzo, Ana Paula Chedid Mendes, Maurício Pimentel, Nadine Clausell, Livia A. Goldraich

**Affiliations:** ^1^ Cardiology Division Hospital de Clínicas de Porto Alegre Porto Alegre, Rua Ramiro Barcelos, 2350, room 2060 Porto Alegre RS 90035‐903 Brazil; ^2^ Post‐Graduate Program in Cardiology and Cardiovascular Sciences, Medical School Universidade Federal do Rio Grande do Sul Porto Alegre Brazil

**Keywords:** Cardiac resynchronization therapy, Exercise‐induced left bundle branch block, Heart failure

## Abstract

Indications of cardiac resynchronization therapy (CRT) do not include exercise‐induced left bundle branch block, but functional impairment could be improved with CRT in such cases. A 57‐year‐old woman with idiopathic dilated cardiomyopathy (ejection fraction 23%) presented with New York Heart Association Class IV and recurrent hospitalizations. During heart transplant evaluation, a new onset of intermittent left bundle branch block was observed on the cardiopulmonary exercise test. CRT was implanted, and 97% resynchronization rate was achieved. In 12 month follow‐up, both clinical and prognostic exercise parameters improved. In patients with heart failure with reduced ejection fraction and no left bundle branch block at rest, exercise test can uncover electromechanical dyssynchrony that may benefit from CRT.

## 1 Introduction

Exercise‐induced left bundle branch block (EI‐LBBB) is rarely observed in exercise testing being associated with worsening symptoms during exertion and adverse prognosis.^1^ Cardiac resynchronization therapy (CRT) is an established therapy for patients with heart failure with reduced ejection fraction and resting left bundle branch block (LBBB).^2^ Potential benefits of biventricular pacing in patients who develop LBBB during exercise are uncertain, and current guidelines do not provide recommendations for resynchronization therapy in this situation. Herein, we present a case of advanced heart failure with EI‐LBBB in which CRT played a major role for clinical improvement.

## 2 Case report

**Figure 1:**
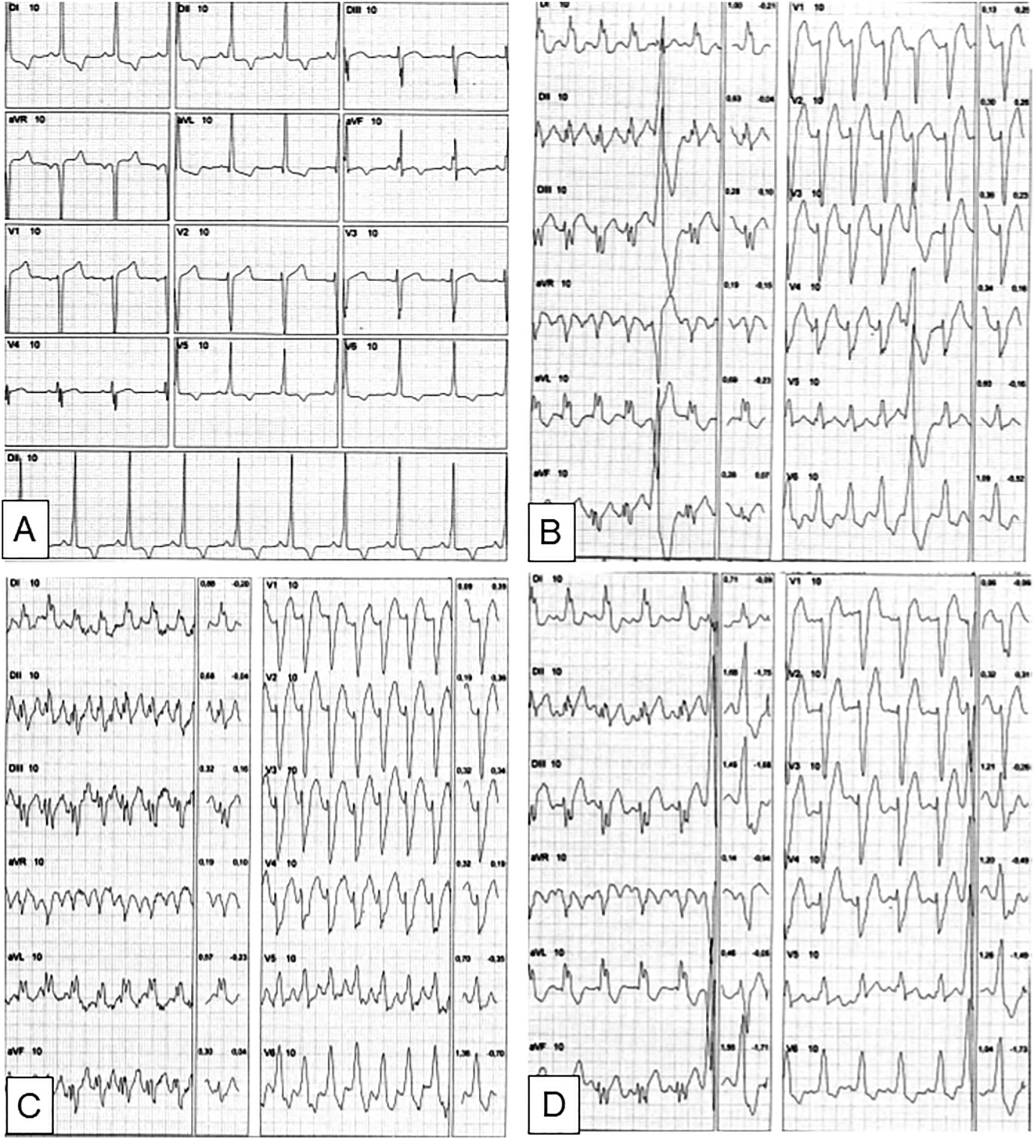
New onset of intermittent left bundle branch block during a cardiopulmonary exercise test. (A) Rest, heart rate (HR) 76 bpm, QRS 80 ms; (B) exercise at 2 min, HR 131 bpm, QRS 200 ms; (C) exercise peak at 7 min, HR 156 bpm, QRS 200 ms; and (D) recovery at 2 min, HR 109 bpm, QRS 200 ms.

A 57‐year‐old woman with a previous 8‐year follow‐up of idiopathic dilated cardiomyopathy presented with worsening functional class and recurrent hospitalizations for heart failure New York Heart Association functional Class IV and Interagency Registry for Mechanically Assisted Circulatory Support Class V. She had been treated with guideline directed medical therapy at maximally tolerated doses—which included enalapril 10 mg b.i.d, metoprolol 50 mg b.i.d., spironolactone 25 mg, furosemide 40 mg b.i.d., and digoxin 0.125 mg daily—for at least 5 years. Comorbidities included active smoking and morbid obesity (body mass index of 37 kg/m^2^). She was mostly limited by exertional fatigue and dyspnoea, despite being mostly euvolemic not dependent on high doses of diuretics. Rest electrocardiogram (ECG) showed sinus rhythm at a rate of 70 bpm and a QRS duration of 80 ms. Left ventricular ejection fraction was 23%, with a dilated left ventricle (end‐diastolic diameter 6.7 cm) and diffuse hypokinesis. A cardiopulmonary exercise testing (CPET) demonstrated severely decreased exercise capacity, with a peak oxygen uptake (VO_2_ peak) of 12 mL/kg/min (or 16.46 mL/kg/min when corrected for lean body mass), along with other variables indicating adverse heart failure prognosis, including minute ventilation/carbon dioxide production (VE/VCO_2_) slope of 42 and periodic breathing. At the second minute of exercise, at a heart rate of 131 bpm, the patient developed LBBB with widening of the QRS to 200 ms. This finding persisted into the recovery phase (*Figure* *1*). Of note, attenuated pressoric response was also observed during the CPET. A subsequent exercise stress echocardiography evidenced exertion‐induced intraventricular and interventricular dyssynchrony, with electromechanical delay from QRS onset to S wave onset from septal, lateral, anterior, and inferior walls of more than 65 ms and difference between left and right pre‐ejection intervals of more than 40 ms, respectively.

As the patient was not considered eligible for cardiac transplant, given her active comorbidities, a decision was made to implant a CRT (Etrinsa 8 HF‐T, Biotronik, Berlim, Germany) to approach the EI‐LBBB. Cardiac magnetic resonance imaging performed prior to CRT insertion showed only a small area of late gadolinium enhancement at the mesocardium basal septum, but no patterns suggestive of specific aetiologies other than non‐ischaemic dilated cardiomyopathy were noted. Initially, biventricular pacing was set at a minimal heart rate of 75 bpm with atrioventricular delay of 105–80 ms, and the ECG showed QRS duration of 145 ms.

**Figure 2:**
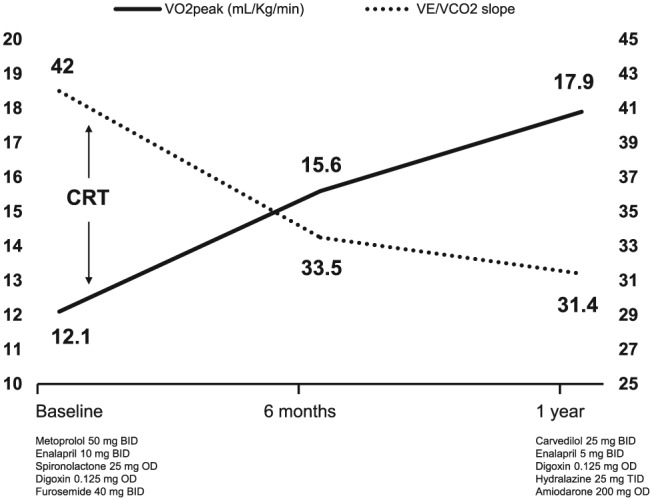
Cardiopulmonary exercise testing variables and medical therapy at baseline and following cardiac resynchronization therapy. CRT, cardiac resynchronization therapy; VO_2_ peak, peak oxygen uptake; VE/VCO_2_, minute ventilation/carbon dioxide production.

Subsequent to CRT implant, the patient improved to New York Heart Association II, and no recurrent heart failure hospitalizations occurred. Medical therapy was relatively unchanged after CRT, except for the introduction of amiodarone due to ventricular arrhythmias and a change from metoprolol to carvedilol and introduction of hydralazine, both to enhance hypertension management, as blood pressure levels increased and tolerance to medications improved after CRT. Pacemaker interrogation showed 94% atrial pacing and 100% biventricular pacing. A few months later, repeat CPET at 6 and 12 months demonstrated significant and sustained amelioration in exercise capacity, as depicted in *Figure* *2*. Biventricular pacing occurred at 100% of the exercise time, and periodic breathing was absent on both exams. Reverse cardiac remodelling and improvement in ejection fraction were also noticed (*Table* 1).

**Table 1:** Echocardiographic parameters following cardiac resynchronization therapy

	Baseline	6 months	1 year
Left atrium (mm)	43	44	46
Left atrium volume index (mL/m^2^)	22	NA	31
Left ventricle end diastole (mm)	67	66	62
Left ventricle end systole (mm)	60	58	57
Ejection fraction (%)	23	23	30
Right ventricular systolic pressure (mmHg)	30	25	19
Tricuspid annular plane systolic excursion (cm)	2.1	NA	1.9

Ejection fraction calculated by Simpson's method. NA, not available.

## 3 Discussion

To the best of our knowledge, this is the first report of CRT implantation in a patient with advanced heart failure and severe exercise limitation attributed to EI‐LBBB. In the present case, CRT was associated with sustained functional capacity and prognostic markers improvement. The patient received at least 5‐year guideline‐based treatment, and no major changes in medications were performed after CRT. This observation highlights the relevance of exercise evaluation in patients with advanced heart failure to better understand their symptomatology, which may be related to the broadening of the QRS complex during exertion; in fact, this patient was severely limited under minimal activities, the exercise test uncovered a EI‐LBBB. Both exercise stress ECG and echocardiography can unravel dynamic electromechanical dyssynchrony that may eventually benefit from CRT.

Exercise‐induced LBBB is a rare condition reported only in 0.5% in patients who underwent exercise testing and is associated with twofold increase in all‐cause mortality 1, 3 In most cases, it is associated with underlying structural heart disease, particularly coronary artery disease.^1^, ^4^ Earlier studies suggested that coronary artery disease was more prevalent when EI‐LBBB onset was observed with lower heart rates (<125 bpm).^4^, ^5^ Our patient developed EI‐LBBB at a heart rate of 131 bpm but had normal coronary arteries. We speculate that the presence of exercise‐induced LBBB was consequential to her underlying dilated cardiomyopathy and contributed to heart failure progression. Although LBBB‐induced cardiomyopathy could be also sought, our patient had no LBBB noted at rest nor did present the diagnostic criteria recently proposed by Sanna et al..^6^

CRT is known to promote left ventricular reverse remodelling. In the Resynchronization Reverses Remodelling in Systolic Left Ventricular Dysfunction trial, which evaluated left ventricular remodelling after CRT, the average absolute improvement in ejection fraction was 3.6% in a follow‐up of 1 year.^7^ Left ventricular end‐systolic and end‐diastolic volumes decreased by 27 and 29 mL, respectively. In a similar follow‐up period, our patient's ejection fraction increased by 7% absolute points, while left ventricular end‐systolic and end‐diastolic volumes decreased by 20 and 37 mL, respectively.

Left ventricular dyssynchrony during exercise occurs in a third of patients with heart failure with reduced ejection fraction being associated with poor outcomes.^2^, ^8^, ^9^ Although the Echocardiography Guided Cardiac Resynchronization Therapy trial showed no benefit of CRT in patients with ejection fraction lower than or equal to 35% and evidence of left ventricular dyssynchrony by echocardiogram, the inclusion criteria was a QRS of 130 ms or less.^10^ By refining CRT guiding modalities, a small trial evidenced that patients randomized to optimization of atrioventricular and interventricular intervals utilizing both Doppler and 3D echocardiography improved ejection fraction and systolic dyssynchrony index to a larger extent when compared with traditional ECG‐based CRT optimization protocols.^11^ Those findings suggest that targeting dyssynchrony markers may be beneficial, irrespective of changes in the QRS complex duration.^11^

There are few published reports of CRT for the management of cardiac symptoms related to EI‐LBBB. Prystowsky et al. reported a case in which severe shortness of breath was coincident with LBBB development at a heart rate of 140 bpm, in the absence of evidence of ischemia or systolic dysfunction. She was initially treated with beta blockers, but there was significant improvement in symptoms only after CRT.^12^ Zeppenfeld et al.^13^ described a case of acute heart failure induced by EI‐LBBB that was refractory to medical therapy and also improved with CRT. Interestingly, there are also reports of EI‐LBBB which improved without CRT. Tanaka et al. published about a male patient with left ventricular ejection fraction of 35% that presented with EI‐LBBB at 100 bpm, associated with significant cardiac dyssynchrony during exertion. The patient was treated with carvedilol and candesartan for 5 months, and the ejection fraction improved to 49% with LBBB occurring only at a heart rate of 126 bpm.^14^ Anderson et al. described a case of a female patient with no structural heart disease and EI‐LBBB at a heart rate of 112 bpm. The patient was treated with cardiac rehabilitation for 3 months. Her symptoms improved and EI‐LBBB appeared only at a higher heart rate of 150 bpm.^15^ In the present report, the clinical improvement after CRT was remarkable. The presence of left ventricular dyssynchrony by echocardiogram may have contributed to the CRT response. Device implant was associated with increased ventricular extrasystolic activity which was successfully managed with beta blocker up‐titration and amiodarone, not interfering in the progressive benefit observed, which included reverse remodelling and improved ejection fraction.

In conclusion, although EI‐LBBB is a relatively rare finding, it should be actively sought in patients with heart failure, even in those with more advanced structural and functional abnormalities. While there is no guideline‐oriented indication for CRT in EI‐LBBB, it could be considered in selected individuals with advanced heart failure with the aim to improve symptoms, particularly in those with left ventricular dyssynchrony.

## Conflict of interest

None declared.
